# Changes in muscle–tendon unit length–force characteristics following experimentally induced photothrombotic stroke cannot be explained by changes in muscle belly structure

**DOI:** 10.1007/s00421-021-04729-x

**Published:** 2021-06-01

**Authors:** Arjun Paudyal, Hans Degens, Guus C. Baan, Wendy Noort, Mark Slevin, Erwin van Wegen, Gert Kwakkel, Huub Maas

**Affiliations:** 1 Department of Life Sciences, Research Centre for Musculoskeletal Science and Sports Medicine, Manchester Metropolitan University, Manchester, UK; 2 Department of Human Movement Sciences, Faculty of Behavioural and Movement Sciences, Vrije University Amsterdam, Amsterdam Movement Sciences, Amsterdam, The Netherlands; 3 Institute of Sport Science and Innovations, Lithuanian Sports University, Kaunas, Lithuania; 4 Department of Rehabilitation Medicine, Amsterdam UMC, Vrije Universiteit Amsterdam, Amsterdam Movement Sciences, Amsterdam Neuroscience, de Boelelaan 1117, Amsterdam, The Netherlands; 5 Department of Physical Therapy and Human Movement Sciences, Feinberg School of Medicine, Northwestern University, Chicago, IL USA; 6 Department of Neurorehabilitation, Amsterdam Rehabilitation Research Centre, Reade, Amsterdam, The Netherlands

**Keywords:** Length–force characteristics, Muscle mechanics, Muscle morphology, Photothrombotic stroke model, Myofascial force transmission

## Abstract

**Purpose:**

The aim of this study was to assess the effects of experimentally induced photothrombotic stroke on structural and mechanical properties of rat *m. flexor carpi ulnaris*.

**Methods:**

Two groups of Young-adult male Sprague–Dawley rats were measured: stroke (*n* = 9) and control (*n* = 7). Photothrombotic stroke was induced in the forelimb region of the primary sensorimotor cortex. Four weeks later, muscle–tendon unit and muscle belly length–force characteristics of the *m. flexor carpi ulnaris*, mechanical interaction with the neighbouring *m. palmaris longus*, the number of sarcomeres in series within muscle fibres, and the physiological cross-sectional area were measured.

**Results:**

Stroke resulted in higher force and stiffness of the *m. flexor carpi ulnaris* at optimum muscle–tendon unit length, but only for the passive conditions. Stroke did not alter the length–force characteristics of *m. flexor carpi ulnaris* muscle belly, morphological characteristics, and the extent of mechanical interaction with *m. palmaris longus* muscle.

**Conclusion:**

The higher passive force and passive stiffness at the muscle–tendon unit level in the absence of changes in structural and mechanical characteristics of the muscle belly indicates that the experimentally induced stroke resulted in an increased stiffness of the tendon.

## Introduction

Stroke is the leading cause of disability and fourth leading cause of adult death; the number of people living with stroke in Europe is expected to increase by one million between 2015 and 2035, reaching 4,631,050 (Sacco et al. 2013; Feigin et al. 2017; Norrving et al. 2018; GBD 2016 Neurology Collaborators 2019) Around 79% of patients suffering a stroke survive for at least 1 year (Radisauskas et al. 2019), but the majority of survivors experience motor disabilities and sensorimotor deficits, including disrupted motor control and spasticity, which have a negative impact on the quality of life of stroke survivors (Burvill et al. 1997; Langhorne et al. 2011).

The motor disabilities are largely a consequence of muscle weakness due, at least transiently, to an impaired cortico- and reticulospinal control of muscles after a stroke (Patten et al. 2004), that contribute to spasms (Bethoux 2015) and limited fascicle shortening (Son et al. 2020). In line with this, it has been observed in a rat model with spastic paresis that the muscle weakness is also accompanied by a narrowing of the length–force relationship and an increased passive stiffness of the muscle–tendon unit (MTU) (Olesen et al. 2014). It was suggested that this increased stiffness could, among other factors, be attributable to loss of number of sarcomeres in series, a situation indeed seen in the paretic biceps brachii of stroke patients (Adkins et al. 2020). This loss of sarcomeres in series is expected to cause a shift of the optimum length to shorter muscle and fascicle lengths. Yet, if anything a lower, rather than higher, force was seen at shorter muscle length in stroke patients (Ada et al. 2003). However, no change in the length–force relationship was observed in single muscles in a rat stroke model (Dormer et al. 2009). Also, spastic muscle fibres develop passive tension at shorter sarcomere length and have a higher elastic modulus compared to the normal muscle fibres (Fridén and Lieber 2003).

A possible explanation for this paradox between shortening of the muscle without a change in the length–force relationship is that muscles mechanically interact with each other via connective tissue linkages at their interface (Maas and Sandercock 2010; Maas 2019). These mechanical interactions are also responsible for the finding that changes in length in one muscle result in changes in force of an adjacent muscle that did not change in length (Olesen et al. 2014). It is hitherto unknown, however, whether stroke induces any changes in the connective tissue linkages between muscles and, hence, the mechanical interaction between muscles. Only one study tested this hypothesis on a small number (*n* = 5) of stroke patients using ultrasound imaging (Diong and Herbert 2015), but direct measurements of force transmission between spastic-paretic muscles have not been performed following stroke.

Another factor to consider is that passive resistance to movement of the MTU is not only a function of the stiffness of the muscle belly, but is also determined by stiffness of the tendon and intramuscular connective tissues (Morse et al. 2008). In fact, it has been observed that the stiffness of the Achilles tendon, but not the length, is reduced in stroke patients (Gao et al. 2009; Zhao et al. 2015; Dias et al. 2019) and this, all else staying the same, would result in a reduced, rather than increased passive stiffness of the MTU. It could thus be that the tendon adaptations compensate, at least partly, for any increase in muscle stiffness. In addition, the similar length of the tendon (Dias et al. 2019), in the face of a reduced number of sarcomeres in series (Adkins et al. 2020), would imply that each sarcomere is at a greater length. The stretched sarcomeres could explain the higher resistance to lengthening without any need to postulate increased muscle tissue stiffness. It therefore remains to be seen whether there is indeed any increase in muscle tissue stiffness after stroke, and whether stroke-induced changes in tendon properties and connective tissue content contribute to the increased passive stiffness of the MTU, or whether the increased stiffness is solely attributable to changes in the muscle fibres. It also needs to be seen whether the muscles are indeed shortened after stroke.

The aim of this study was to assess the effects of experimentally induced stroke on structural and mechanical properties of rat *m. flexor carpi ulnaris* (FCU). Specifically, we measured the MTU and muscle belly length–force characteristics of FCU, mechanical interaction with the neighbouring *m. palmaris longus* (PL), the number of sarcomeres in series within muscle fibres, and the physiological cross-sectional area (PCSA) four weeks after a photothrombotic stroke.

## Materials and methods

### Animals

Two groups of young-adult male Sprague–Dawley rats were measured: 4 weeks post-stroke (*n* = 9) and a control group (*n* = 7). One animal (body mass 350 g) had to be euthanized 1 week post-stroke, because of an ear infection, and was hence only used for assessment of the infarct (see below). Body mass at the time of this experiment (i.e., 4 weeks post-stroke) was 465 ± 29 g for the stroke group (*n* = 8) and 488 ± 65 g for the control group, which was not significantly different (*p* = 0.404).

Surgical and experimental procedures were according to guidelines and regulations concerning animal welfare and experimentation set by Dutch law and approved by the Committee on Ethics of Animal Experimentation at the VU University Amsterdam (Permit Number: FBW 12-01). All animals were euthanized after the experimental series with an overdose of intracardially injected pentobarbital sodium followed by a double-sided pneumothorax.

### Surgical procedures for experimental stroke

We used the photothrombotic model of stroke that induces a local cortical infarct via photo activation of an intravenously injected (saphenous vein) photoactive dye (Watson et al. 1985; Hoff et al. 2011). In brief, when the injected dye is illuminated by a cold light, the photochemical reaction leads to endothelial damage, activation of the platelets, and thrombosis, that together result in an interrupted local blood flow.

Rats were anaesthetized through inhalation of isoflurane (induction 4%, maintenance 1.5–2%). The lowest possible dose of maintenance anaesthesia was set as judged by the complete absence of withdrawal reflexes. Preoperative, each animal received a single dose of painkiller buprenorphine (intraperitoneally; 0.01 mg/kg body mass; Temgesic; Schering-Plough, Maarssen, The Netherlands). After shaving the scalp and ventral hindlimb region, lidocaine (2%) was injected subcutaneously at the incision site on the head. The head of the rat was fixed in a stereotaxic frame in a prone position. Body temperature was constantly monitored and maintained at 37 °C using a heating pad. Under aseptic conditions, a midline incision of 2.0–2.5 cm was made through the scalp and the skin was retracted laterally to expose the skull surface with the help of retractors. After dissecting the connective tissues from the skull, the periost was gently removed and dried with the help of a cauteriser to identify the coronal and sagittal sutures. Once the bregma and lambda were exposed, the illumination area was defined as 1.5–4.5 mm lateral and + 4.0 mm to − 4.0 mm anterior/posterior to the bregma. This area corresponds to the forelimb region of the primary sensorimotor cortex (Paxinos and Watson 2006). An optic fibre bundle connected to a halogen light source (Schott KL 1500 LCD, Germany) with a green filter (wavelength 560 nm) was positioned in close contact with the skull surface. All remaining exposed area of the skull was then covered with black tape to prevent undesired illumination.

An incision was made on the ipsilateral hindlimb to expose the saphenous vein. Before illumination, the photosensitive dye Rose Bengal (Sigma-Aldrich) solution (25 mg/kg body mass) was infused into the saphenous vein at a rate of 5.625 mg/min via a single syringe infusion pump (World Precision Instrument, SP100IZ) using a black catheter. Within 2 min, the light was turned on for illumination. After 20 min of illumination, the light exposure was stopped, and both the limb and skull incisions were closed. After termination of isoflurane anaesthesia, the condition of the rats was monitored for 2 h before they returned to their home cage.

Rats were observed daily after the stroke induction. At day 3 post-stroke, the vibrissae-evoked forelimb placing test was performed to confirm stroke induction (Schallert 2006). For all rats, stroke impaired placement of the contralateral forelimb on the tabletop when the contralateral vibrissae came in contact with the table. Rats were still able to perform feeding, drinking, and grooming activities.

### Surgical procedures for assessment of muscle mechanical properties

Before surgery and data collection, rats were anesthetized by an intraperitoneal injection of urethane (initial dose 1.2 ml/100 g body mass, 12.5% urethane solution), according to standard procedures in our laboratory (Tijs et al. 2016). If withdrawal reflexes were present, additional doses of 0.2 ml were administered. Throughout the surgery and measurements, body temperature was monitored and maintained at approximately 37 °C using a heating pad. The exposed muscles were regularly irrigated with isotonic saline solution to prevent dehydration.

The surgical procedures have been described in more detail elsewhere (Maas and Huijing 2009). Briefly, the distal tendons of *m. flexor carpi ulnaris* (FCU) and *m. palmaris longus* (PL) were identified and dissected free from the surrounding tissue leaving the connective tissue surrounding the muscle bellies intact. The exposed muscles were regularly irrigated with isotonic saline solution to prevent dehydration. The wrist was held in the neutral position (i.e., 180° flexion, 0° ulnar deviation, 0° pronation) and the elbow joint at approximately 90°. This position was referred to as reference length (L_ref_). Markers (i.e., knots with 7–0 suture, Ethicon) were placed on the distal tendons of the PL and FCU, and to assess changes in muscle belly length (*L*_m_), a marker was placed at the most distal end of FCU muscle belly.

The PL tendon was cut from the insertion site and Kevlar thread was tied for attachment to the force transducer. The FCU tendon was excised along with the pisiform bone from the carpus leaving the tendon-to-bone connection intact, which was attached to Kevlar thread. Further dissection was performed in the brachial compartment to secure a metal clamp to the humerus for later fixation in the experimental setup. Within the brachial compartment, the ulnar and median nerves were identified and cut from the brachial plexus in the axilla. The nerves were prevented from dehydrating by covering them with a thin piece of latex. A custom-made bipolar cuff electrode was placed on the ulnar and median nerves. The musculocutaneous nerve was cut, denervating the biceps brachii and brachialis muscles.

### Experimental setup and protocol

**Fig. 1:**
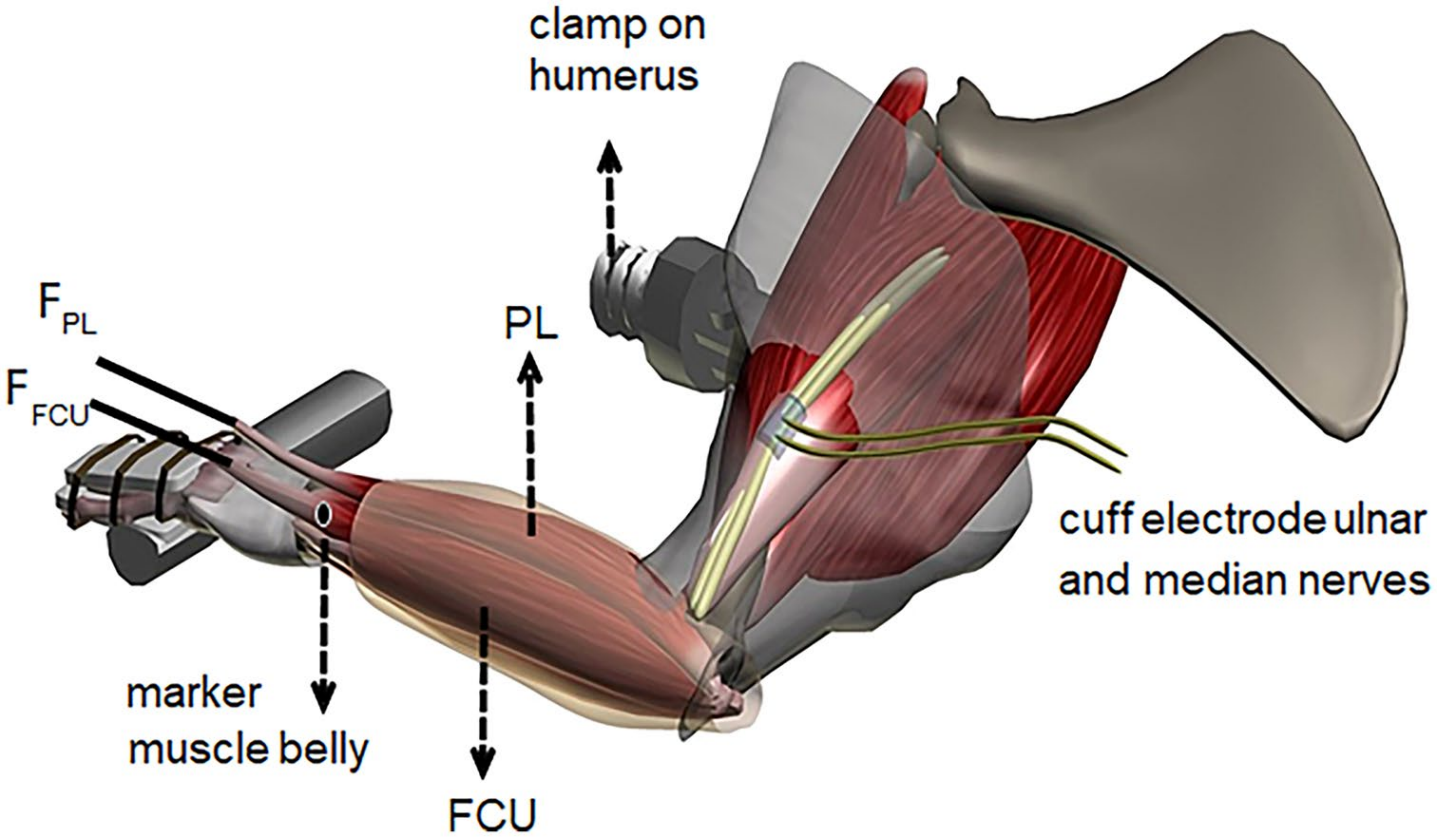
Schematic view of experimental setup. The distal tendons of the m. flexor carpi ulnaris (FCU) and m. palmaris longus (PL) were connected to two separate force transducers (F_FCU_, F_PL_) mounted on micro-positioners. Connective tissue surrounding the muscle bellies was left intact. A bipolar cuff electrode was placed on the ulnar and median nerves. The forelimb was secured rigidly by clamping the humerus with a metal clamp as well as by firmly tying the dorsal side of the paw to a metal plate. The palmar side of the manus faced upwards, the wrist was kept at neutral position (i.e. 180° flexion, 0° ulnar deviation, 0° pronation) and the elbow joint at 90°. In the experiment, the length of PL was kept constant at reference length

After placing the rat on a heating pad, the forelimb was secured rigidly by clamping the humerus and firmly tying the dorsal side of the manus to an aluminium plate with 1–0 silk suture. The forearm was secured in the horizontal position with the palmar side of the manus faced upwards, the wrist in neutral position as described above. With the use of the Kevlar thread in series with steel rods, each distal tendon was connected to two separate force transducers (BHL Electronica, Canton, Massachusetts, USA; maximal output error < 0.1%, compliance of 0.0162 mm/N) (Fig. 1).

The muscles were maximally activated by supramaximal stimulation of the ulnar and median nerves (amplitude ~ 0.5 mA, pulse width 100 µs) via the cuff electrode connected to a constant current source (Digitimer DS3, Digitimer Ltd., Hertfordshire, England). Two twitches (interstimulus interval 300 ms) were evoked followed 300 ms later by a tetanic contraction (stimulation frequency 100 Hz) (Maas and Huijing 2009). Force data were collected at 1 kHz using a custom-built DC strain gauge amplifier (400–1600×) and an A/D converter (PCI-6221; National Instruments, Austin, TX, USA). To remove any history effects of muscle contraction at high lengths, FCU was excited alternately at high and low lengths repeatedly until the forces output at low lengths were reproducible (Huijing and Baan 2001). For all experimental conditions, PL length was kept at reference length. Isometric forces of FCU and PL were measured at various muscle–tendon unit lengths of FCU (*L*_MTU_). The MTU was lengthened distally with 0.5 mm increments starting at active slack length (i.e., zero active force) to approximately 1 mm over optimum length (*L*_opt_). To minimize muscle fatigue, after each contraction the FCU was released to a low length and rested for 2 min. A custom-made data acquisition program was used to control stimulation of the nerves and for data collection and storage.

In five out of eight rats with stroke and for five out of eight control rats, the position of the muscle–tendon junction marker was recorded at each muscle–tendon unit length (*L*_MTU_) using a digital camera (25 frames/s, DCR‐ TRV6E, Sony) to obtain muscle belly lengths (*L*_m_) before and during contraction at each MTU length. The muscle belly length of the other rats could not be assessed, due to the technical limitations of the experimental setup.

### Data analysis

Passive and active forces were extracted from the measured force–time series. Passive force was assessed by calculating the mean of the force output for 50 ms between the second twitch and tetanic contraction (MATLAB, version 2014b, Mathworks). Total force was assessed by calculating the mean for the last 100 ms of the tetanic plateau. Active force was assessed by subtracting passive force from total force at the same MTU length. Active force as a function of MTU length was fitted by a polynomial using stepwise regression. The curve fit was determined by increasing the order of the polynomial (up to maximally a sixth order). Passive force as a function of MTU length was fitted to a double exponential function

1 $$f\left( x \right)\, = \,a.e^{b.x} \, + \,c.e^{d.x} \left( {R^{2} \, > \,0.99} \right).$$

Passive stiffness of the MTU, calculated as the slope of the fitted passive length–force curve, was assessed at *L*_opt_.

At each MTU length, muscle belly lengths (*L*_M_) before and during contraction were obtained from the recorded videos. To calculate the active forces at each muscle belly length, the passive forces were subtracted from total forces, both assessed at the same muscle belly length. Both active and passive forces as a function of muscle belly length were fitted as described for MTU length. Passive stiffness of the muscle belly, calculated as slope of the fitted passive length–force curve, was assessed at *L*_opt_.

All passive and active forces at each MTU and muscle belly length for each rat were then normalized to their respective estimated physiological cross-sectional area (PCSA; see below).

### Collection of morphological and histological data

After completion of mechanical measurements, FCU muscle was excised, weighed, and cut longitudinally into two halves. One half was fixed in formaldehyde solution [3.7% formaldehyde; 16% ethanol; and 1.67 g/L thymol] for at least 2 weeks at room temperature and the other half was snap-frozen in liquid nitrogen and stored at − 80° for further analysis.

Estimation of the PCSA was performed using measurements of muscle density and optimum fibre length (Huijing and Maas 2016). Muscle density (ρm) was calculated according to

2 $$\begin{array}{ccccc} \rho m{\mkern 1mu} & = {\mkern 1mu} {\rm{Muscle mass in air}}/\left( {{\rm{Muscle mass in air}}} \right.\\ & \quad \left. { - {\rm{ Mass of water displaced by muscle}}} \right) \end{array}$$

Three full-length fascicles were removed from distal, intermediate, and proximal regions of the muscle belly. Three intact single muscle fibres were dissected from each fascicle and mounted on microscope slices. Mean sarcomere length was then assessed by counting the number of sarcomeres in series within 200 μm samples at approximately 15 locations along the length of the fibre. The serial number of sarcomeres (#sarc) within muscle fibres was calculated by dividing fibre length, as measured directly on images of isolated muscle fibres (ImageJ), by mean sarcomere length. This value was used to assess optimum fibre length (*L*_fao_)

3 $$L_{{{\text{fao}}}} \left( {{\mu m}} \right)\, = \,\# {\text{ Sarc }} \times \, L_{{{\text{sao}}}} .$$

A value *L*_sao_ = 2.4 μm was used as the optimum sarcomere length (*L*_sao_) for FCU (Huijing and Maas, 2016). Muscle density and optimum fibre length were used to estimate the PCSA

4 $${\text{PCSA}}\, = \,{\text{Mass}}/({\text{Density}} \times L_{{{\text{fao}}}} ).$$

### Confirmation of infarct

To confirm the infarct, 2,3,5-Triphenyl-2H-Tetrazolium Chloride (TTC) staining was performed to differentiate metabolically active and inactive tissues. After completion of mechanical measurements, the brain was cut out and 2 mm-thick slices were obtained using a vibratome (Campden Instruments Ltd, UK). Slices were put in a small jar with 2% TTC in a water bath (37 °C) for 20 min to obtain pink colour with white infarct area. After 20 min, slices were fixed in 4% paraformaldehyde overnight at 4 °C.

### Statistics

Statistical analyses were performed using IBM SPSS version 22. A two-way repeated-measures ANOVA was performed to examine the group differences in length–force curves (within-subject factor: MTU/muscle belly length; between-subject factor: group) with MTU or muscle belly length as the repeated measurements factor. Greenhouse–Geisser correction was applied in all ANOVAs, because the sphericity was violated. If a significant interaction was found, two-tailed t tests were performed to identify differences in active force, passive force, and passive stiffness between groups at *L*_opt_.

A repeated-measures two-way ANOVA was used also to test for differences in number of sarcomeres in series between groups and location-related differences (i.e., distal, intermediate, and proximal), and group × location interactions of the fibre. The unequal variance type was used if the highest SD was twice the lowest SD; else, the equal variance type was used. All values are presented as mean ± SD and level of significance was set at *p* < 0.05.

## Results

### Confirmation of infarct

**Fig. 2:**
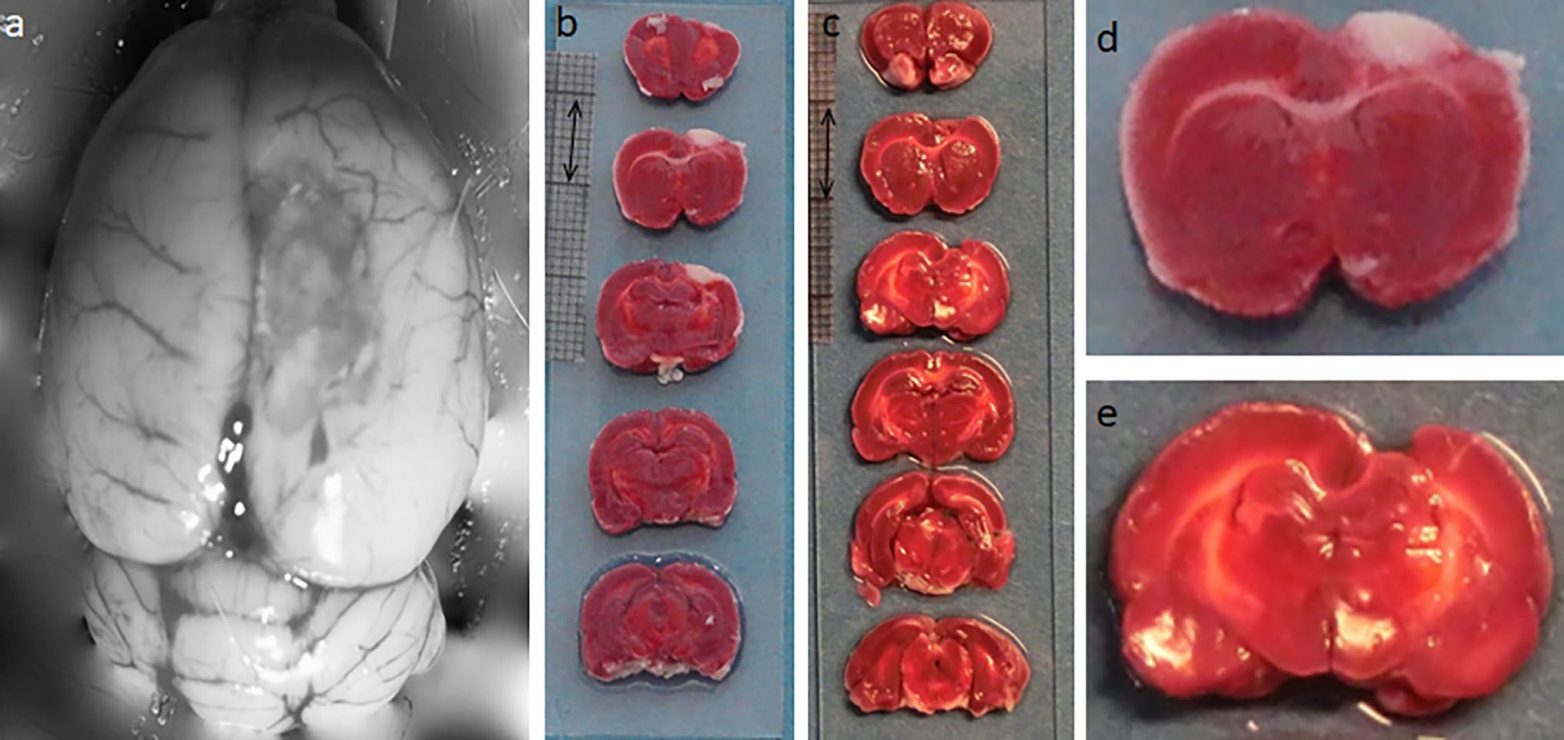
Photographs of ischemic infarct in the brain after photothrombotic stroke. **a** Representative photograph of the brain 4 weeks after stroke, superior view. **b**, **d** TTC (2,3,5-Triphenyl-2H-Tetrazolium Chloride) stained coronal sections of the brain from a rat euthanised 7 days after induction of stroke; this rat was not used for length–force assessment. **c**, **e** TTC stained coronal sections of the brain 4 weeks after stroke from a representative rat also tested for muscle mechanical properties. Double arrow indicates 10 mm

The photothrombotic ischemia resulted in an infarct of the target brain area in all rats (Fig. 2). One rat was euthanised after 1 week and a clear region of metabolically inactive tissue was observed in the brain (Fig. 2b, d). Four weeks after stroke, this region appeared to consist of gel-like tissue (Fig. 2a), which was washed away during the procedures for the TTC staining (Fig. 2c, e).

### MTU length force characteristics of FCU muscle

**Fig. 3:**
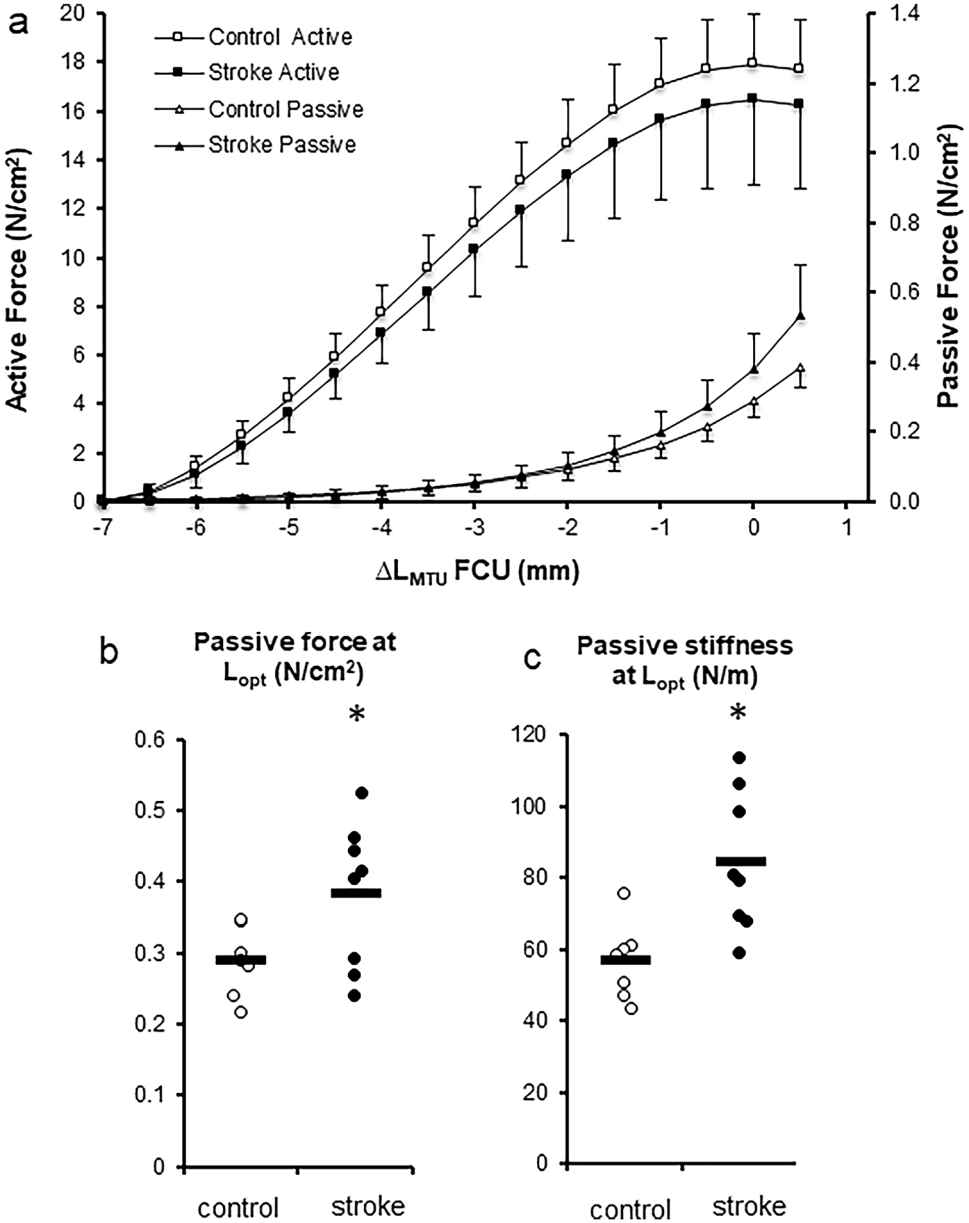
Length–force characteristics of flexor carpi ulnaris (FCU muscle). **a** Active and passive forces are plotted as a function of muscle–tendon unit (MTU) length. MTU length is expressed as the deviation from the length at which maximal active force is generated (∆*L*_MTU_). For passive and active forces, a significant effect of MTU length (*p* < 0.001) was found. **b** Passive forces at optimum length (*L*_opt_). Passive force at optimum length (*L*_opt_) was higher in the stroke than in the control group (*p* = 0.045). **c** At *L*_opt_, passive MTU stiffness was higher in stroke than in control group (*p* = 0.006). *Significant difference (*p* < 0.05). Optimal length occurs at ‘0’ on the *x* axis. Values are mean ± SD (*n* = 7 and *n* = 8 for control and stroke group, respectively)

For both control and stroke rats, the FCU showed the classical length–force characteristics (Fig. 3a). For both passive and active forces, a significant effect of MTU length (*p* < 0.001 for both) but no differences between groups were found (*p* = 0.196 and *p* = 0.261, respectively). For passive forces, a significant length × group interaction was observed (*p* = 0.022). There was no such interaction for active forces (*p* = 0.427). Passive force at optimum length (*L*_opt_) was higher in the stroke than in the control group (*p* = 0.045; Fig. 3b). At *L*_opt_, passive MTU stiffness was significantly higher in stroke than in control group, *p* = 0.006, Fig. 3c). These results indicate that passive forces increase to a greater extent when increasing MTU length in the stroke than in the control group.

### Muscle belly length–force characteristics of FCU muscle

**Fig. 4:**
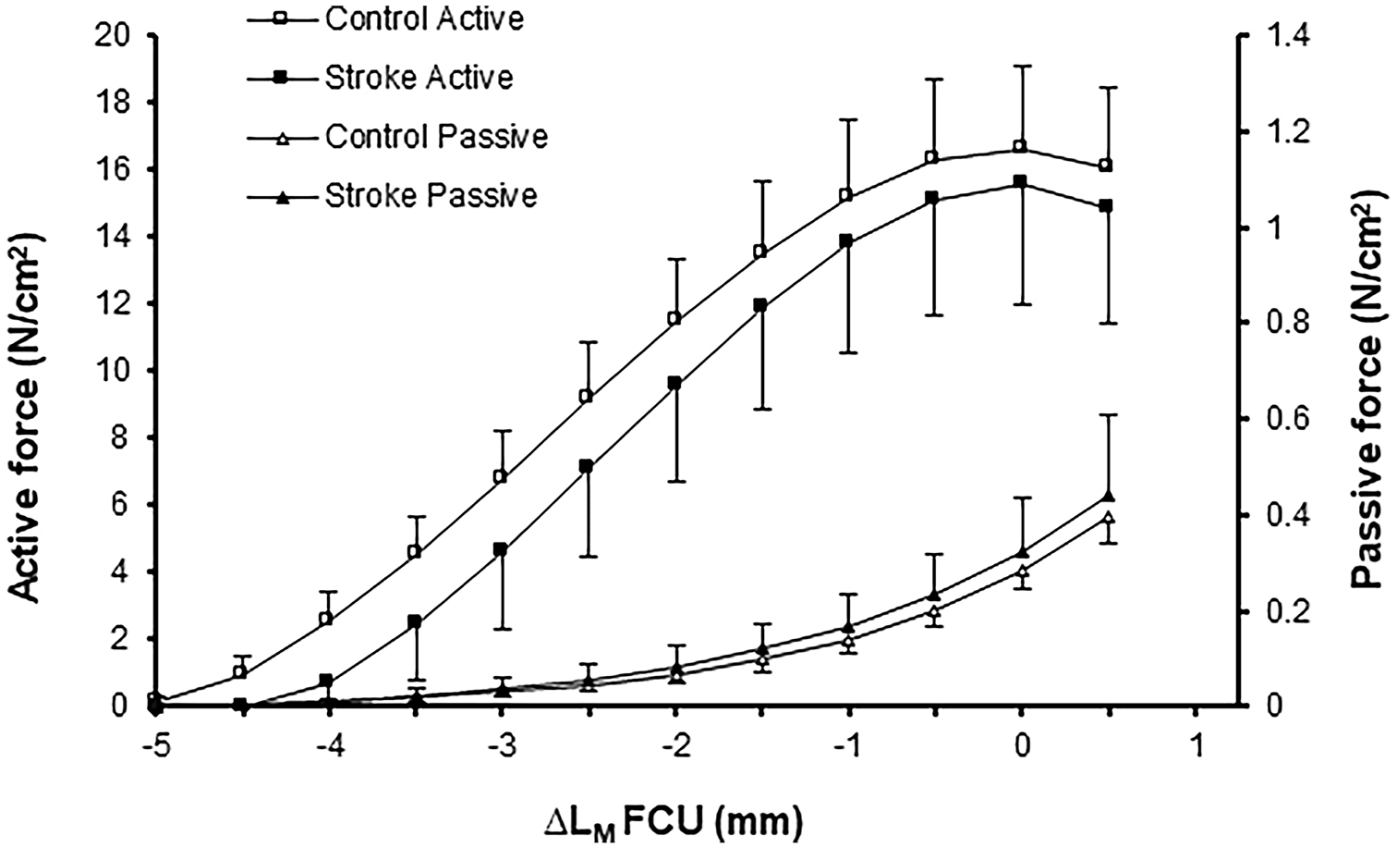
Muscle belly length–force characteristics of FCU muscle. Active and passive forces are plotted as a function of muscle belly length. Muscle belly length is expressed as the deviation from the length corresponding maximal active force (∆*L*_M_). Group (active *p* = 0.239, passive *p* = 0.495) and length (*p* < 0.001) effects were found, as interaction (length × group, active *p* = 0.551, passive *p* = 0.625)**.** Optimal length occurs at ‘0’ on the *x* axis. Values are mean ± SD (*n* = 5 and *n* = 5 for control and stroke group, respectively)

For both passive and active forces, ANOVA indicated a muscle belly length effect (*p* < 0.001, for both), but no group effect (*p* = 0.495 and *p* = 0.239, respectively) or significant interaction effects (*p* = 0.625 and *p* = 0.551, respectively) were found (Fig. 4). These results indicate that stroke did not cause changes in mechanical characteristics at the muscle belly level.

### Mechanical interaction between FCU and PL muscle

**Fig. 5:**
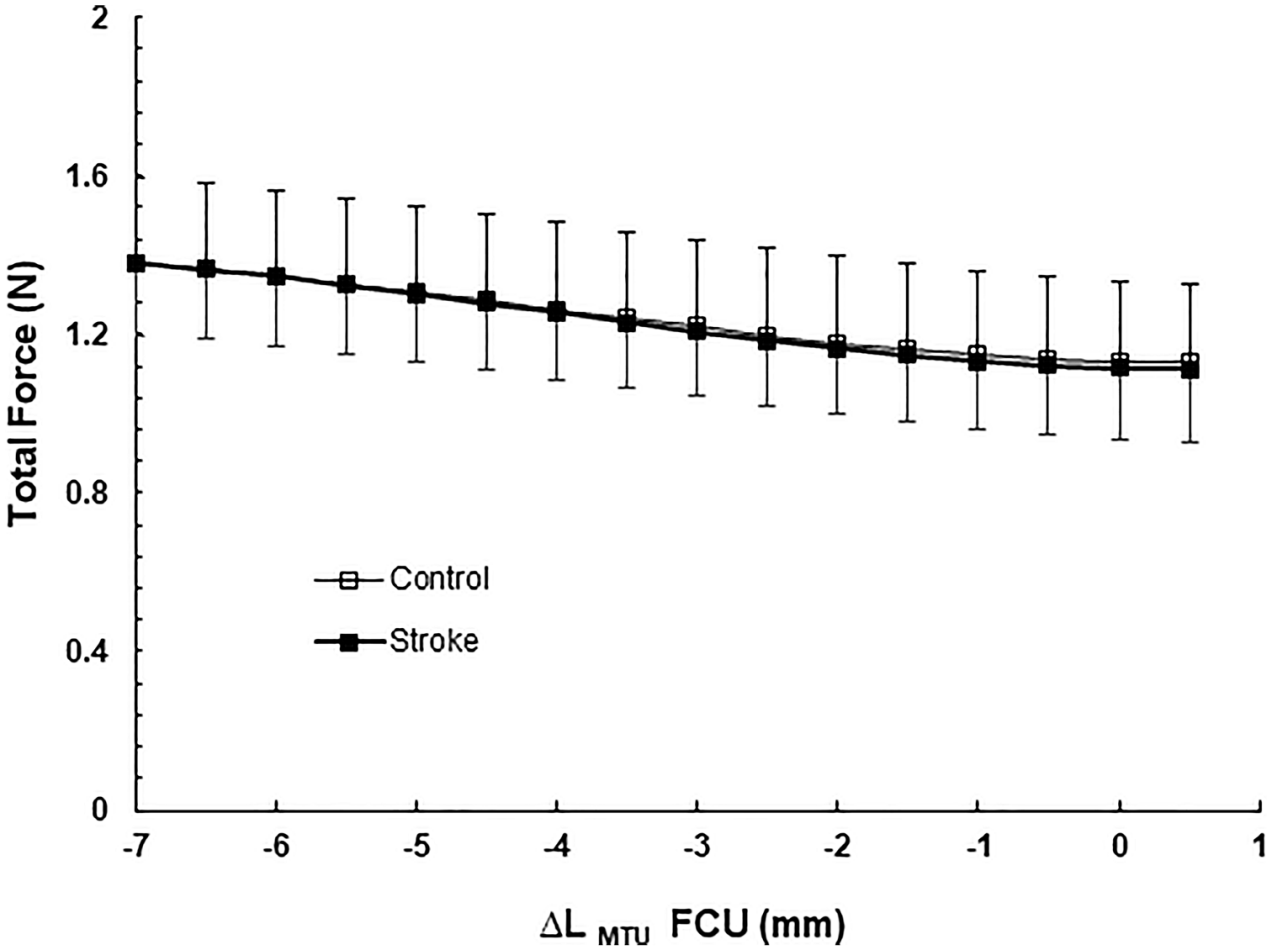
Total forces of the palmaris (PL) muscle plotted as a function of flexor carpi ulnaris (FCU) muscle–tendon unit length (∆*L*_MTU_). ANOVA revealed a significant length effect (*p* = 0.001) on total PL forces, but no group effect (*p* = 0.954), nor an interaction effect (*p* = 0.574). Note that PL was kept at a constant muscle–tendon unit. Values are mean ± SD (*n* = 7 and *n* = 8 for control and stroke group, respectively)

In both groups, increasing the MTU length of FCU resulted in a decrease in force exerted at the distal tendon of PL (Fig. 5). Note that the MTU length of PL was kept constant. ANOVA indicated a significant length effect (*p* < 0.001) on total PL forces, but no group effect (*p* = 0.963), nor an interaction effect (*p* = 0.543). These results indicate significant mechanical interaction between FCU and PL muscles, but no changes in response to stroke.

### FCU muscle morphology

**Fig. 6:**
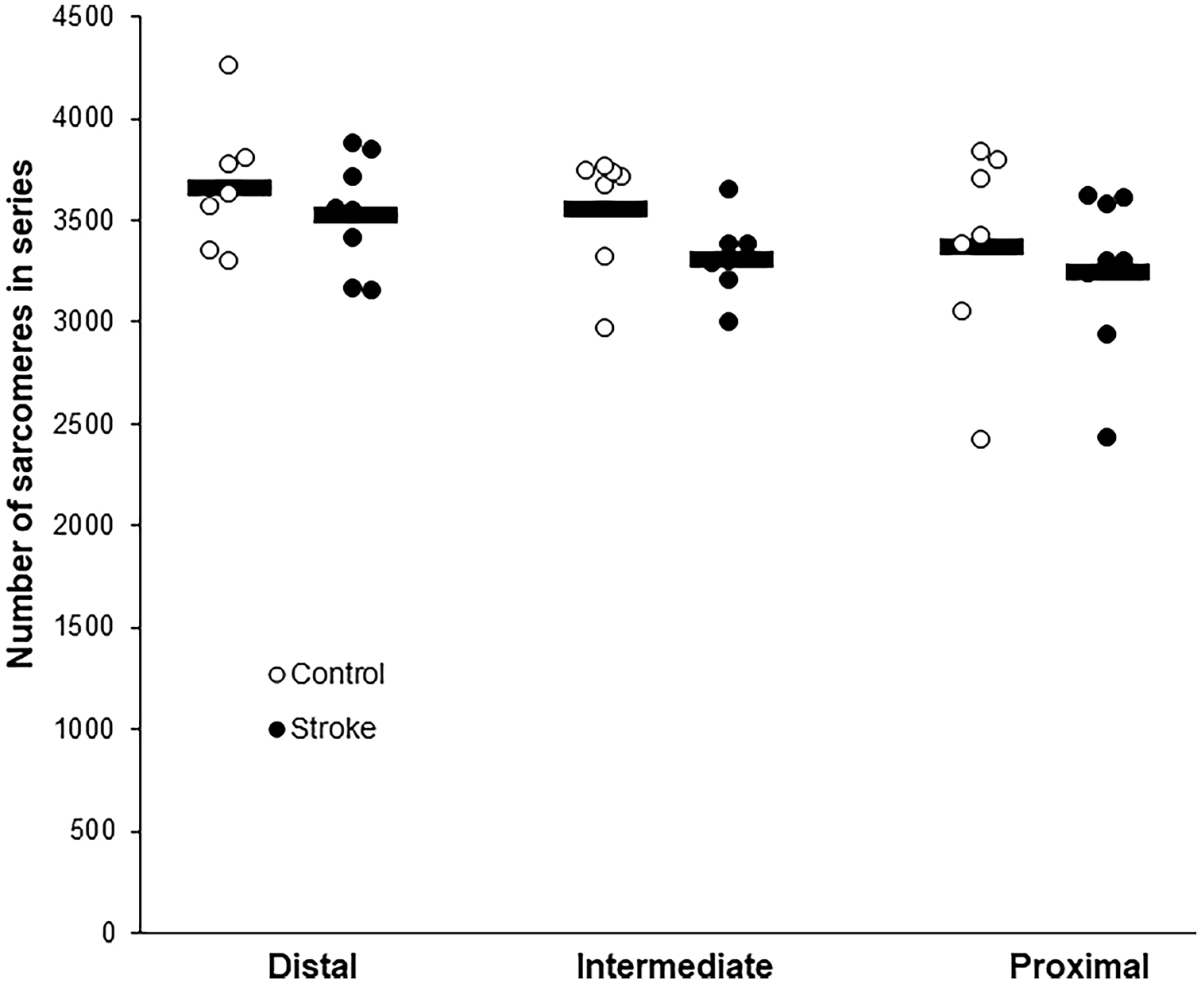
Effects of stroke on serial numbers of sarcomere within distal, intermediate, and proximal regions of flexor carpi ulnaris muscle fibres. Mean and individual data (*n* = 7 for control; *n* = 8 for stroke) of serial numbers of sarcomeres within different regions of FCU muscle fibres. There was no significant main effect of group (*p* = 0.250) and location (*p* = 0.920), nor a significant interaction (*p* = 0.684) on serial sarcomere number

Morphological parameters did not differ between groups (Table 1). No significant main effect of group (stroke vs. control) on serial sarcomere number of FCU muscle fibres was observed (*p* = 0.250), nor an effect of location within the muscle (i.e., proximal, intermediate, or distal; *p* = 0.092). Mean number of sarcomeres in series was 3529 ± 149 for the control group and 3361 ± 149 for the group with stroke. Also, no significant interaction effect between group and location within the muscle was found (*p* = 0.684) (Fig. 6).

**Table 1:** Morphological characteristics of flexor carpi ulnaris muscle

	Control (*n* = 7)	Stroke (*n* = 8)	*p* value
FCU muscle mass (mg)	301 ± 30	289 ± 26	0.422
FCU Density (ρ_m_) (g/cm^3^)	1.055 ± 0.003	1.060 ± 0.017	0.502
PCSA (cm^2^)	0.34 ± 0.04	0.34 ± 0.04	0.968
	(*n* = 5)	(*n* = 5)	
FCU MTU length at *L*_opt_ (mm)	37.2 ± 2.5	37.3 ± 3.4	0.930
FCU muscle belly length at *L*_opt_ (mm)	26.2 ± 0.9	27.5 ± 1.7	0.181
FCU tendon length at *L*_opt_ (mm)	11.0 ± 1.6	9.9 ± 1.7	0.304

Body mass and FCU muscle mass at testing day. Length of FCU muscle belly, tendon, and MTU were estimated from videos of the experimental forelimb with MTUs at optimal length from 5 rats in each group. Data are assessed 4 weeks post-stroke induction for the stroke group and comparable age for control group. Values are means ± SD

*FCU* flexor carpi ulnaris, *PCSA* estimated physiological cross-sectional area, *MTU* muscle–tendon unit, *L*_*opt*_ optimal length

## Discussion

In this study, we assessed the effects of experimental stroke in the forelimb region of the primary sensorimotor cortex on structural and mechanical properties of rat flexor carpi ulnaris muscle. Our main findings are that stroke: (1) resulted in higher passive force and stiffness of the FCU at optimum length, but did not alter (2) the length–force characteristics of FCU muscle belly, (3) the extent of mechanical interaction with neighbouring PL muscle, and (4) muscle morphological characteristics.

The photothrombotic model is one of the least invasive models of stroke induction in small animals, which induces localised photochemical cortical infarcts with a very low mortality rate (Fluri et al. 2015). In the present study, the stroke clearly resulted in damage to the motor cortex (Fig. 2). With high reproducibility, this model can be customized to study the behavioural deficit associated with the targeted area of the brain (Lee et al. 2007). Although this model has been used for many post-stroke behavioural studies, the mechanical properties of the forelimb skeletal muscles have never been investigated. Skeletal muscle properties were evaluated at only one time point, i.e., 4 weeks post-stroke in this study.

Our observations of an absence of significant atrophy and an unaltered active length–force relationship after stroke are in line with previous studies on rats after unilateral focal ischaemic insult (Dormer et al. 2009). However, we did find a substantially higher passive stiffness of the muscle–tendon unit (MTU), something observed previously in muscles from spastic rats (Olesen et al. 2014). In spastic hemiparesis, the increased muscle–tendon unit stiffness and decreased range of motion were particularly attributable to an increase in fascicle stiffness (Zhang et al. 2013). Such an increased fascicle stiffness may be attributable to loss of sarcomeres in series, as seen in the paretic biceps brachii of patients with chronic stroke (Adkins et al. 2020). However, we did not observe any loss of sarcomeres in series. It is plausible that stroke only results in loss of sarcomeres in series in the presence of spasticity. Alternatively, 4 weeks between stroke and measurement may have been too short to induce changes in muscle architecture. However, 4 weeks of immobilization at short MTU length resulted in a substantial (28%) reduction of sarcomeres in series within soleus muscle (Heslinga et al. 1995). In addition, the number of sarcomeres in series 5 weeks after transferring rat FCU muscle to the insertion of the extensor carpi radialis muscle was reduced by 25% (Huijing and Maas 2016). Thus, 4 weeks should be enough time for rat FCU muscle to adapt its number of sarcomeres in series.

Lee et al. (2014) reported that the affected muscles 4 weeks post-photothrombotic stroke in mice showed no apparent spastic behaviour. Although not systematically assessed, we also did not observe any obvious signs of spasticity in the rats in this experiment. Our model thus gave us a unique opportunity to study the effects of stroke without the confounding effects of spasticity. It is possible that the often observed progressively worsening spasticity in stroke patients only becomes evident in rodents at a point later than 4 weeks after stroke. The changes we observed over this period are smaller than those found in muscles of humans post-stroke (Canning et al. 2004). Additional research with multiple time points should be performed to investigate the time-course of changes in muscle mechanical properties after stroke.

While there were no significant stroke-induced changes in the active length–force relationship, our results did show an increased passive force and stiffness of the MTU at optimum length after stroke. The absence of a change in the length–force relationship at the muscle belly level, as well as an unaltered number of sarcomeres in series and PCSA indicates that the higher passive forces and stiffness at the MTU level should be attributed to an increased tendon stiffness. Because we did not place a marker on the distal end of the tendon and tracking the distal end without this marker could not be done with enough accuracy, we could not quantify external tendon stiffness. Whether or not the tendon stiffness was increased in our stroke model, it is in contrast to the reported reduction in the stiffness of the Achilles tendon in stroke patients (Gao et al. 2009; Zhao et al. 2015; Dias et al. 2019). It should be noted that stroke occurs particularly in older adults, where we used young-adult rats. Indeed, aging has been shown to cause a decrease in tendon stiffness in humans (Reeves 2006). On the other hand, mice tendons stiffen with aging (Wood et al. 2011). In addition, a decreased stiffness is an expected adaptation to a period of (partial) muscle paralysis and a chronic reduction in force generation capacity. In contrast, muscle denervation of several ankle plantar flexors in the cat did not result in a reduction in the peak ankle moment, and hence, the forces exerted on the Achilles tendon, as the animals walked with increased ankle dorsi-flexion (Prilutsky et al. 2011).

In conclusion, photothrombotic stroke resulted in higher passive force and stiffness of the FCU at optimal muscle–tendon unit length. Interestingly, stroke did not change the active length–force characteristics of the FCU muscle belly, muscle morphological characteristics including the PCSA and serial number of sarcomeres in FCU muscle. In addition, the extent of mechanical interaction with neighbouring PL muscle was not affected by stroke. Additional research needs to be carried out to determine whether these changes are attributable to an increased tendon stiffness.

## Abbreviations

FCU: *m. flexor carpi ulnaris*
L_fao_: Optimum fibre length
*L*_m_: Muscle belly length
*L*_MTU_: Muscle–tendon unit length
*L*_opt_: Optimum length
*L*_ref_: Reference length
*L*_sao_: Sarcomere length
MTU: Muscle–tendon unit
PCSA: Physiological cross-sectional area
PL: *m. palmaris longus*
TTC: 2,3,5-Triphenyl-2H-tetrazolium chloride
